# Poly (ADP-Ribose) Polymerase 1 Is Required for Protein Localization to Cajal Body

**DOI:** 10.1371/journal.pgen.1000387

**Published:** 2009-02-20

**Authors:** Elena Kotova, Michael Jarnik, Alexei V. Tulin

**Affiliations:** Fox Chase Cancer Center, Philadelphia, Pennsylvania, United States of America; National Institute of Diabetes and Digestive and Kidney Diseases, United States of America

## Abstract

Recently, the nuclear protein known as Poly (ADP-ribose) Polymerase1 (PARP1) was shown to play a key role in regulating transcription of a number of genes and controlling the nuclear sub-organelle nucleolus. PARP1 enzyme is known to catalyze the transfer of ADP-ribose to a variety of nuclear proteins. At present, however, while we do know that the main acceptor for pADPr *in vivo* is PARP1 protein itself, by PARP1 automodification, the significance of PARP1 automodification for *in vivo* processes is not clear. Therefore, we investigated the roles of PARP1 auto ADP-ribosylation in dynamic nuclear processes during development. Specifically, we discovered that PARP1 automodification is required for shuttling key proteins into Cajal body (CB) by protein non-covalent interaction with pADPr *in vivo*. We hypothesize that PARP1 protein shuttling follows a chain of events whereby, first, most unmodified PARP1 protein molecules bind to chromatin and accumulate in nucleoli, but then, second, upon automodification with poly(ADP-ribose), PARP1 interacts non-covalently with a number of nuclear proteins such that the resulting protein-pADPr complex dissociates from chromatin into CB.

## Author Summary

Previous studies revealed vital roles for the Poly(ADP-ribose) Polymerase (PARP) protein in developmental program regulation, as well as in chromatin loosening and transcriptional activation. The main gap in our understanding of PARP protein roles during development involves integrating the biological activities of PARP protein into a general network of nuclear protein dynamics and gene expression. Here, we report the functional association of PARP protein activity with proteins trafficking through Cajal bodies. Cajal bodies (CBs) are nuclear organelles that regulate the biogenesis of RNA-protein complexes involved in transcription and splicing. We demonstrate that (1) PARP is present in CBs, (2) its loss leads to CB disassembly, (3) an increase in PARP levels leads to formation of CBs, and, finally, (4) PARP localization is ecdysone sensitive. We further propose a model in which PARP controls protein trafficking through the CB and contributes to CB formation.

## Introduction

The basic enzymatic reactions catalyzed by PARP1 involve transferring ADPr from NAD to either a protein acceptor or to an existing poly(ADP-ribose) (pADPr) chain [1]. More than thirty pADPr acceptor proteins have been identified. However, multiple observations *in vivo* and *in vitro* have confirmed that the main acceptor of nuclear pADPr *in vivo* is PARP1 protein itself (reviewed in [2]). This suggests that PARP1 automodification plays important roles in the regulation of nuclear functions. To understand these roles, the present study proposes to elucidate the mechanisms of such automodified PARP1 action on nuclear processes.

Most PARP1 protein binds to chromatin and accumulates in nucleoli [3]–[6]. Previously, we showed that local activation of transcription in specific domains of chromatin always correlates with local activation of PARP1 protein enzymatic reaction followed by automodification of PARP1 protein within the same chromatin domains [7],[8]. The automodification of PARP1 causes it to dissociate from the chromatin template and lose its ability to synthesize pADPr [9],[10]. This event, however, allows either the automodified PARP1, or free pADPr, to gain another special biological activity by specifically enabling either one to interact non-covalently with a broad class of nuclear proteins which bear a conserved domain recognizing pADPr [11]–[14]. Thus, automodified PARP1 which accumulates around a particular locus could serve as a local signal for binding and transportation of those proteins, as well as for modification of their biochemical properties. To date, however, no one has shown the functional significance of those interactions *in vivo*.

Poly(ADP-Ribose)Glycohydrolase (PARG) protein is the only known catalyst of pADPr degradation *in vivo* [10],[15],[16]. Recently, we demonstrated that loss of PARG reduces overall PARP1 activity by trapping a large fraction of the PARP1 protein in the automodified state, which is removed from active chromatin and then accumulates in specific nucleoplasmic organelles [10]. These findings raise the possibility that automodified PARP1 targets from chromatin into these organelles and that, consequently, automodified PARP1 could serve as a shuttle to deliver other proteins interacting with pADPr into these organelles. In this paper, we show that those organelles are Cajal bodies (CBs). CBs (also known as “Coiled bodies”) are spherical sub-organelles found in the nucleus of proliferative or metabolically active cells in plant, yeast, and animals. CBs are possible sites of assembly or modification of the transcription machinery of the nucleus [17]. Since PARP1 protein also controls transcription and nucleoli and since automodified PARP1 is targeted into CB, PARP1 could be an important link in this process. Moreover, CBs are often seen attached to the nucleolus and share many nucleolar protein components, such as protein Fibrillarin [18] and p80 protein coilin which is a common specific marker of CBs [19] that we have used in our experiments.

Here, we show that PARP1 protein interacts with key components of CBs and that the presence of pADPr is critical for their delivery to CB. Specifically, our data show that chromosomal PARP1 molecules become activated by developmental cues, such as ecdysteroid signaling, and are automodified by pADPr. Automodified PARP1 then binds protein components of nucleoli and chromatin. Following that, automodified PARP1 serves as a “shuttle” for protein delivery to Cajal bodies for recycling, thus contributing to protein trafficking through CBs and to the overall stability of Cajal body.

## Results

### PARP1 Protein Is an Obligatory Resident of Cajal Bodies

**Figure 1:**
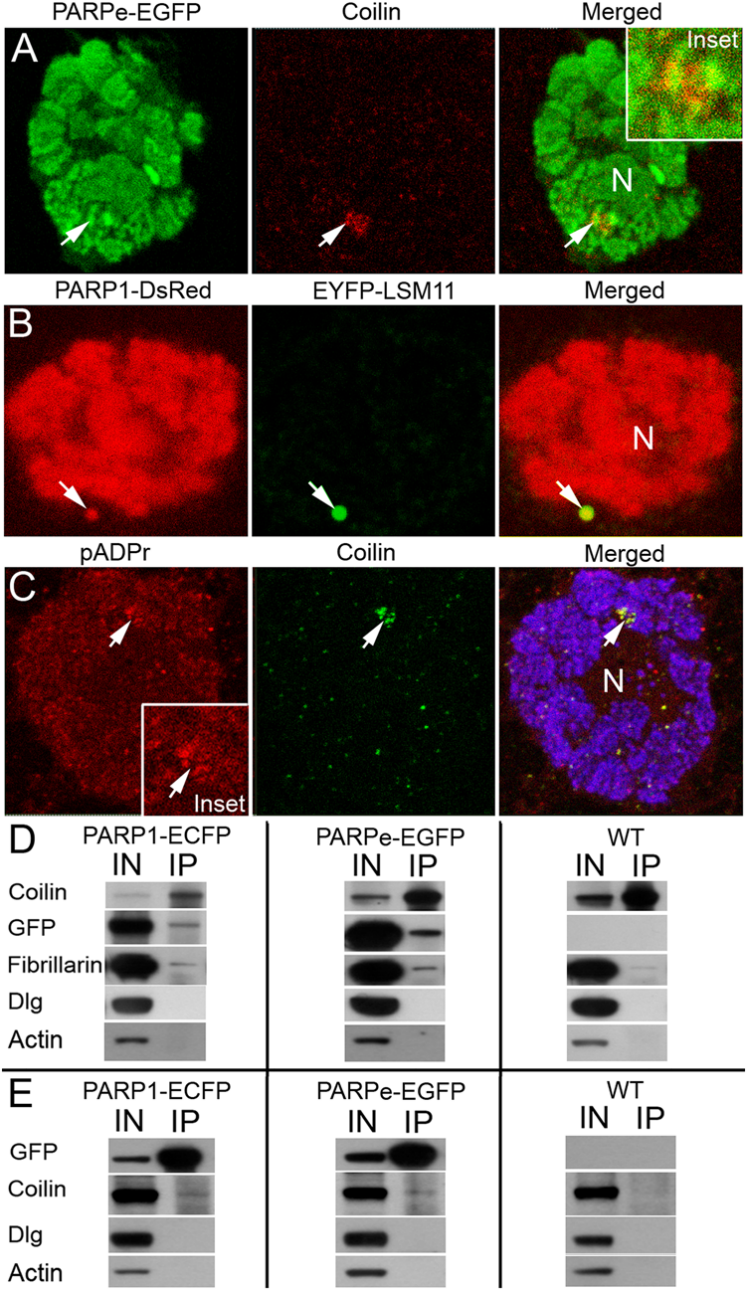
PARP protein is localized to Cajal body (CB) and histone locus body (HLB) and interacts with HLB/CB components. N indicates nucleolus. A. The dissected salivary glands expressing PARPe-EGFP (green) transgenic construct were sectioned to ultra thin sections and stained with rabbit anti-Coilin antibody (red). Position of HLB/CB is indicated with arrow. Inset. Magnification of the chromatin block containing CB is shown. B. The dissected salivary glands co-expressing PARP1-DsRed (red) and EYFP-LSM11 (green) transgenic constructs were stained with the DNA binding dye Draq5 (blue). Position of HLB/CB is indicated with arrow. C. The dissected salivary glands from wild-type larvae were fixed and sectioned, followed by immunostaining with rabbit anti-pADPr (Red); Guinea Pig anti-Coilin antibody (green) and mouse anti-histone H1 (blue). Position of CB/HLB is indicated with arrow. Accumulation of pADPr in CB is clearly shown. Inset. Magnification of the chromatin block containing CB is shown. D. Immunoprecipitation assays using Guinea Pig anti-Coilin antibody. *Drosophila* stocks expressing PARP1-ECFP and PARPe-EGFP were used. Wild type *Drosophila* stock was used as a control. To detect protein on Western blots, the following antibodies were used: rabbit anti-Coilin; rabbit anti-GFP (to detect PARP1-ECFP and PARPe-EGFP); rabbit anti-Fibrillarin; mouse anti-Dlg; and mouse anti-Actin. E. Immunoprecipitation assays using rabbit anti-GFP antibody. *Drosophila* stock expressing PARP1-ECFP and PARPe-EGFP were used. Wild type *Drosophila* stock was used as a control. To detect protein on Western blots, the following antibodies were used: Guinea Pig anti-Coilin; mouse anti-GFP (to detect PARP1-ECFP and PARPe-EGFP); mouse anti-Fibrillarin; mouse anti-Dlg; and mouse anti-Actin.

To study PARP1 protein nuclear localization during *Drosophila* development, we used *Drosophila* stock ubiquitously expressing PARP1-DsRed recombinant protein. Previously, we biologically validated this construct by testing its ability to rescue a *Parp^CH1^* mutation phenotype and by using immunofluorescence to assess recombinant protein localization to chromatin [6],[8]. We also demonstrated that the expression level of PARP1-DsRed transgene does not exceed the level of endogenous PARP1 expression [5]. In wild-type nuclei, the automodified PARP1 protein form is represented by a very minor and transient fraction of the total PARP1 protein. Therefore, at all stages of development and in all tissues, most of the PARP1 protein pool is shown to be associated with chromatin. However, using high resolution laser confocal microscopy, we often observed small PARP1-positive (Figure S1A) and pADPr-positive extrachromosomal particles (Figure S1B), the structural properties, size and localization of which all suggested certain similarities to Cajal bodies (CBs) [20] and the related organelle, histone locus body (HLB) [21],[22]. In order to test this hypothesis, we compared localization of the Coilin protein, which is a classical marker of CBs and HLBs [19], and snRNP-specific protein LSM11, which has been reported as a histone locus body (HLB) marker in *Drosophila* [23], with localization of recombinant PARP protein. Wild-type nuclei typically showed single CB and single HLB [22], while PARP protein demonstrated colocalization with LSM11- and Coilin-positive particles in nuclei of et least 20 salivary glands taken for analysis (Figure 1A, B). Moreover, co-immunostaining of wild-type *Drosophila* tissues demonstrated strong colocalization of pADPr and Coilin protein (Figure 1C) Taken together, these observations suggest that PARP1 protein, as well as poly(ADP-ribosyl)ated proteins, are residents of CB and HLB in *Drosophila*.

Next, in order to test interaction of PARP1 protein with other CB residents, we performed co-immunoprecipitation experiments using antibody against *Drosophila* Coilin protein (gift from Joe Gall) to precipitate Coilin-containing protein complexes. We prepared total protein extracts using *Drosophila* PARP1 mutant stock ubiquitously expressing PARP1-ECFP or PARPe-EGFP transgenic constructs. First, we found that PARP1-ECFP, as well as PARPe-EGFP, proteins always co-purify with Coilin, as well as Fibrillarin protein (Figure 1D), which has also been reported as a component of CBs [18]. Additional co-immunoprecipitation experiments with anti-Fibrillarin antibody confirmed the specificity of PARP1-Fibrillarin interaction (Figure S2A). Moreover, it was found that cytoplasmic disk-large (Dlg) and Actin proteins, which were used as a negative control, did not interact with Coilin (Figure 1D).

Second, using antibody against GFP, we precipitated PARP1-ECFP and PARPe-EGFP, which contain protein complexes, and we detected Coilin protein on Western blots, while neither Dlg nor Actin proteins were observed (Figure 1E).

To confirm the observed interaction, we next performed control experiments. First, to test whether GFP protein itself interacts with CB, we immunoprecipitated protein complexes with anti-GFP antibody from *Drosophila* stock expressing nuclear GFP protein (Figure S2B). No interaction between GFP and Coilin proteins has been detected. Second, we demonstrated that preimmune antibodies do not co-immunoprecipitate PARP protein as well as components of CB and HLB (Figure S2C), thus giving additional support to the finding that interactions of PARP1 protein with components of CB and HLB are specific.

To confirm that intrinsic PARP1 protein interacts with proteins of CB, as well as ectopically expresses, we performed a co-immunoprecipitation experiment using antibody against the C-terminal domain of PARP1 (generous gift of Dr. W. Lee Kraus). A significant amount of Coilin protein was co-purified with PARP1 from wild-type *Drosophila* (Figure S2D), while the same experiment performed with anti-GFP antibody demonstrated no Coilin protein co-purification. Taken together, these results indicate that PARP1 protein is a component of CB and HLB and that PARP1 protein physically interacts with components of CB *in vivo*.

### PARP1 Protein Controls Localization of Proteins in CBs

**Figure 2:**
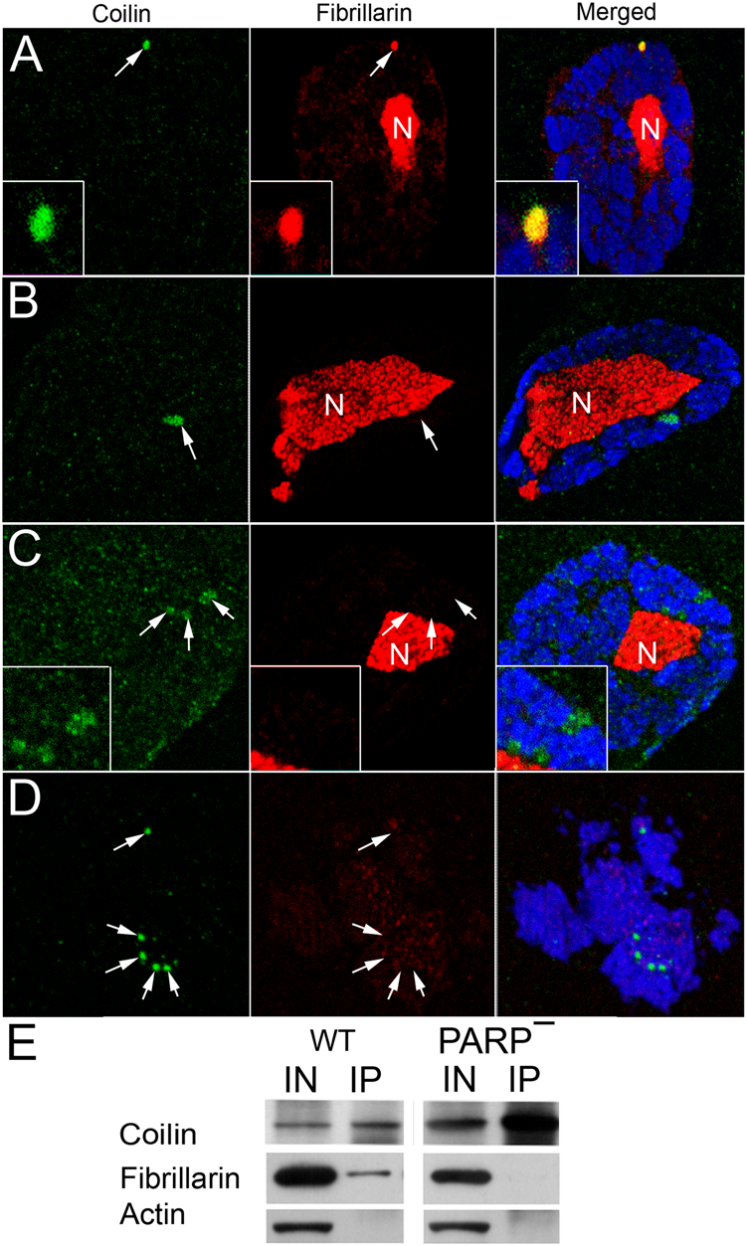
PARP1 protein controls Cajal body integrity. The following antibodies were used for immunostainings: Guinea Pig anti-Coilin (green); rabbit anti-Fibrillarin (red); and the DNA binding dye Draq5 (blue). N – nucleolus. The position of CB is indicated with arrow. Wild-type (A) and PARP1 mutant (B, C, D) salivary glands were used for immunostaining. E. Immunoprecipitation assays using mouse anti-Coilin antibody. The total protein extracts from wild-type (WT) and PARP1 mutant (PARP^−^) flies were used. To detect protein on Western blots, rabbit anti-Coilin, rabbit anti-Fibrillarin, and mouse anti-Actin antibodies were used.

Previously, we demonstrated that PARP1 protein is involved in chromatin loosening and transcriptional activation as well as the establishment and maintenance of nucleolus [6]. Nucleolar and CB proteins, such as Fibrillarin, relocated from nuclei to cytoplasm in *PARP1* mutant cells [6]. In order to test whether PARP1 protein is required for CB function, we compared localization of typical CB resident proteins, Fibrillarin and Coilin, in wild-type flies and *PARP1* mutants. In wild-type animals, Coilin protein localizes exclusively in CB (Figure 2A). Fibrillarin protein occupies CB as well as nucleoli (Figure 2A). Fibrillarin and Coilin completely co-localized in the CB (Figure 2A) and demonstrated physical interaction in co-immunoprecipitation experiments (Figure 2E). *Drosophila PARP1* mutants survive until maternally-contributed Parp1 mRNA and PARP1 protein degrade [6]. The earliest phenotype which we detected in those mutants is the relocation of Fibrillarin from CBs (Figure 2B). At the same time, Fibrillarin still occupies the nucleolus, and CB is detected as a single intact organelle (Figure 2B). At the later stages, however, we detected the breakdown of single CB into disintegrating Coilin-positive particles (Figure 2C). Finally, even at the most severe *PARP1* mutant stage, when Fibrillarin relocalized from nucleolus to cytoplasm, we were still able to identify a few small, separated Coilin-containing bodies in nuclei (Figure 2D). Co-immunoprecipitation experiments using antibodies against Coilin confirmed the absence of interaction between Coilin and Fibrillarin in the *PARP1* mutant animals (Figure 2E). We conclude from these data that PARP1 protein is necessary for interaction of key protein components of CB and may, therefore, also be essential for CB.

### Mutating Parg Induces Additional HLB/CBs in Nucleoplasm

**Figure 3:**
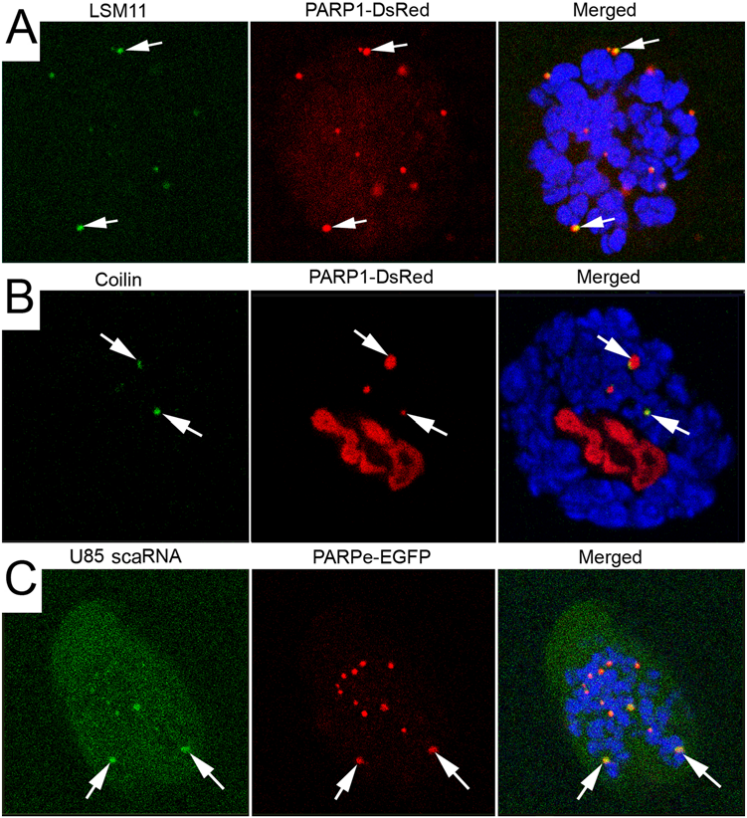
The inhibition of PARG function induces ectopic CBs and relocation of PARP1 protein from chromatin into CBs. (A, B) The salivary glands dissected from the *Parg^27.1^* mutants expressing PARP1-DsRed (red) transgene. DNA was stained with Draq5 dye. Antibody against LSM11 protein (green) (A) and anti-Coilin (green) (B) were used to detect Cajal bodies. N – nucleolus. (C) Fluorescent *in situ* hybridization of U85 scaRNA. The salivary glands dissected from the *Parg^27.1^* mutants expressing PARPe-EGFP transgene. U85 scaRNA probe (green) detects CBLP. Rabbit antibody against GFP protein (red) detects PARPe-EGFP. To visualize chromatin (blue), mouse anti-histone H1 antibody was used. Arrows indicate CBLP with strong colocalization of U85 scaRNA and PARP protein.

Although we have seen that intact unmodified PARP1 protein is dynamically associated with chromatin [5], it is also true that activation and automodification lead to dissociation of PARP1 from chromatin [5],[9],[10]. Therefore, we suggest that extrachromosomal PARP1, which shuttles into HLB/CB, as indicated above, is automodified. In wild-type animals, only a tiny transient fraction of PARP1 protein is automodified due to the presence of PARG protein, which rapidly recycles pADPr [10],[16]. As a consequence, comparative analysis of pADPr quantity in wild-type is ineffective. Therefore, we used the *parg^27.1^* mutant background because the pADPr-modified form of PARP1 protein is stabilized in this setting. To detect HLBs and CBs, we again used LSM11 and Coilin proteins. The number and sizes of HLBs and CBs significantly increased in *parg^27.1^* mutant nuclei (average number of additional bodies is twelve), and the HLB/CB markers are always colocalized with PARP1-DsRed protein (Figure 3A, B). To test whether other key components are also localized to ectopic CB/HLB, we performed fluorescent *in situ* hybridization (FISH) using a U85 scaRNA probe. Using the U85 scaRNA probe demonstrated perfect colocalization with PARP-positive particle in *parg^27.1^* mutant tissues (Figure 3C). These results suggest that the accumulation of proteins modified with pADPr, or automodified PARP1 protein alone, induces formation of additional HLB/CBs in the *parg^27.1^* mutant nuclei. It is then important to ask which signals drive PARP1 protein activation and resultant relocation. In order to address this question, we performed analysis of PARP1-DsRed protein nuclear dynamics from the perspective of *Drosophila* development.

**Figure 4:**
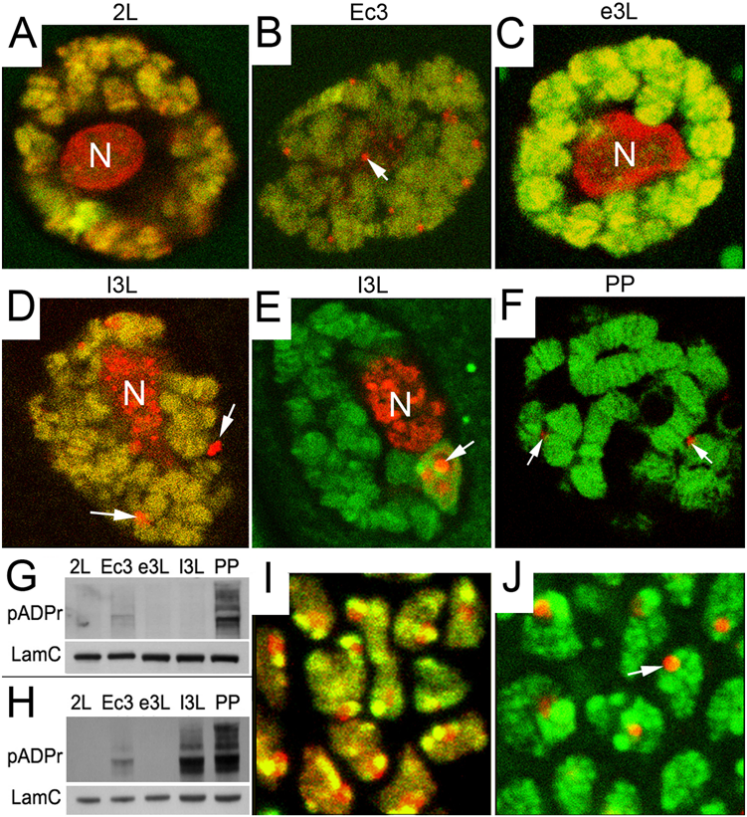
Ecdysteroid hormone controls PARP1 protein localization and activation *in vivo*. Localization of PARP1-DsRed (red) protein was analyzed by confocal microscopy in live *Parg^27.1^* mutant *Drosophila* tissues. DNA was stained with Draq5 reagent (green). PARP-containing nucleoplasmic bodies are indicated with arrows. Salivary gland nuclei in late second-instar larvae (A), in ecdysis III (B), in early third-instar (C), late third-instar (D, E), and late prepupae (F) are shown. (G–H) Western blot analysis of the pADPr accumulation in wild- type (G) and *Parg^27.1^* mutant animals (H) during development is shown. Mouse anti-pADPr antibody was used. Mouse antibody against Lamin C was used as a loading control. (I–J) The comparison of the PARP1-DsRed protein localization in diploid tissues (wing imaginal disk) of wild-type (I) and *Parg^27.1^* mutant (J), late third-instar larvae, is shown.

Under certain circumstances [16], *Drosophila Parg* mutants survive up to adult stage. Therefore, all developmental steps are available for analysis. In the *parg^27.1^* mutant nuclei, we found that PARP1 protein is exclusively associated with chromatin until late second-instar larvae (Figure 4A). However, at the next step of development, ecdysis III, 60% of nuclei demonstrate the appearance of extrachromosomal PARP1-positive particles (Figure 4B). This same developmental stage demonstrates severe elevation of pADPr by Western blotting (Figure 4G–H). After progressing through ecdysis, in early third-instar larvae, the level of pADPr is decreased (Figure 4G–H), and no nucleoplasmic PARP1 particles are detected (Figure 4C). The most dramatic accumulation of pADPr is detected in late third-instar and early prepupae (Figure 4G–H). Those same stages in PARG mutants (100% of nuclei) are characterized by progressive relocation of the PARP1 protein from chromatin to nucleoplasmic bodies (Figure 4D–E). These results suggest that PARP1 protein activation and its relocation to HLB/CBs are events linked and sensitive to ecdysteroid signaling and transcriptional activation cascades which accompany this signaling. In late prepupae of Parg mutant cells, no PARP1 protein could be detected in chromatin (Figure 4F), which suggests that, without PARG, the automodified PARP1 could not be recovered and probably degrades through a protein degradation pathway. Diploid tissues of the *parg^27.1^* mutant also demonstrate PARP1 protein relocalization from chromatin into HLB/CBs (Figure 4I–J) in a manner similar to the phenotype described above for polyploid tissue. However, only one HLB/CB-like organelle could be seen in the diploid nucleus (Figure 4J; Figure S4C). Therefore, the effect on PARP1 activation by ecdysteroid and PARP1 relocation into nucleoplasmic bodies is not tissue-specific, but general for all tissues. Next, we performed electron microscopic analysis of *Parg* mutant tissues which do not express PARP1-DsRed recombinant protein. We did not find any difference in either the number, or ultrastructure (Figure S4), of nucleoplasmic particles between the *parg^27.1^* mutant nuclei alone (Figure S4B) or the *parg^27.1^* mutant expressing PARP1-DsRed recombinant protein (not shown), indicating that ectopic PARP1-DsRed expression does not modify *parg^27.1^* mutant phenotypes. We next tested whether other components of CB also localize into ectopic CBs in Parg mutants. We found that subunits of RNA Polymerase II accumulate in those bodies (Figure S5A–B).

Thus, our data, as summarized above, demonstrate that accumulation of pADPr and dynamic relocation of PARP1 protein from chromatin to HLB/CBs correlate directly with two waves of ecdysteroid secretion which control *Drosophila* development: ecdysis III and metamorphosis. This, in turn, suggests that ecdysteroid-dependent transcriptional activation is a process which both involves PARP1 protein enzymatic activation and drives PARP1 automodification.

### Ecdysone Controls Induction of Ectopic HLB/CBs and PARP1 Protein Relocalization into HLB/CB In Vitro

To confirm that ecdysteroid signaling is responsible for PARP1 protein dynamic relocation, we analyzed its effect on dissected salivary glands treated with a physiological concentration of ecdysone (20E). We dissected salivary glands from third-instar larvae of wild-type and *parg^27.1^* mutant, both expressing PARP1-DsRed protein. We had chosen the stages of development before induction of PARP1 nucleoplasmic bodies in the *parg^27.1^* mutant. Dissected *Drosophila* tissues were cultured, as described in [24], on a slide with depression in dark humid camera with and without 20E. After 3–4 hours, PARP1-DsRed protein localization was analyzed by confocal microscopy. Consistent with our observations *in vivo*, 20E treatment does not affect PARP1 localization in wild-type nuclei (Figure S6A). However, induction of extrachromosomal PARP1-DsRed-positive particles was detected in *parg^27.1^* mutant nuclei after 20E treatment (Figure S6B). Under the same conditions, untreated *parg^27.1^* salivary glands did not show PARP1-DsRed protein relocation to the nucleoplasm (Figure S6C). These results are highly reproducible and were observed in all 20 dissected samples. Our observations show that accumulation of pADPr and shuttling of PARP1 protein into HLB/CBs are, therefore, under the control of ecdysteroid hormone signaling during development of the fruit fly.

### The Expression of PARG-EGFP Transgenic Construct in Parg mutants Suppresses the Shuttling of PARP1 Protein into HLB/CBs

**Figure 5:**
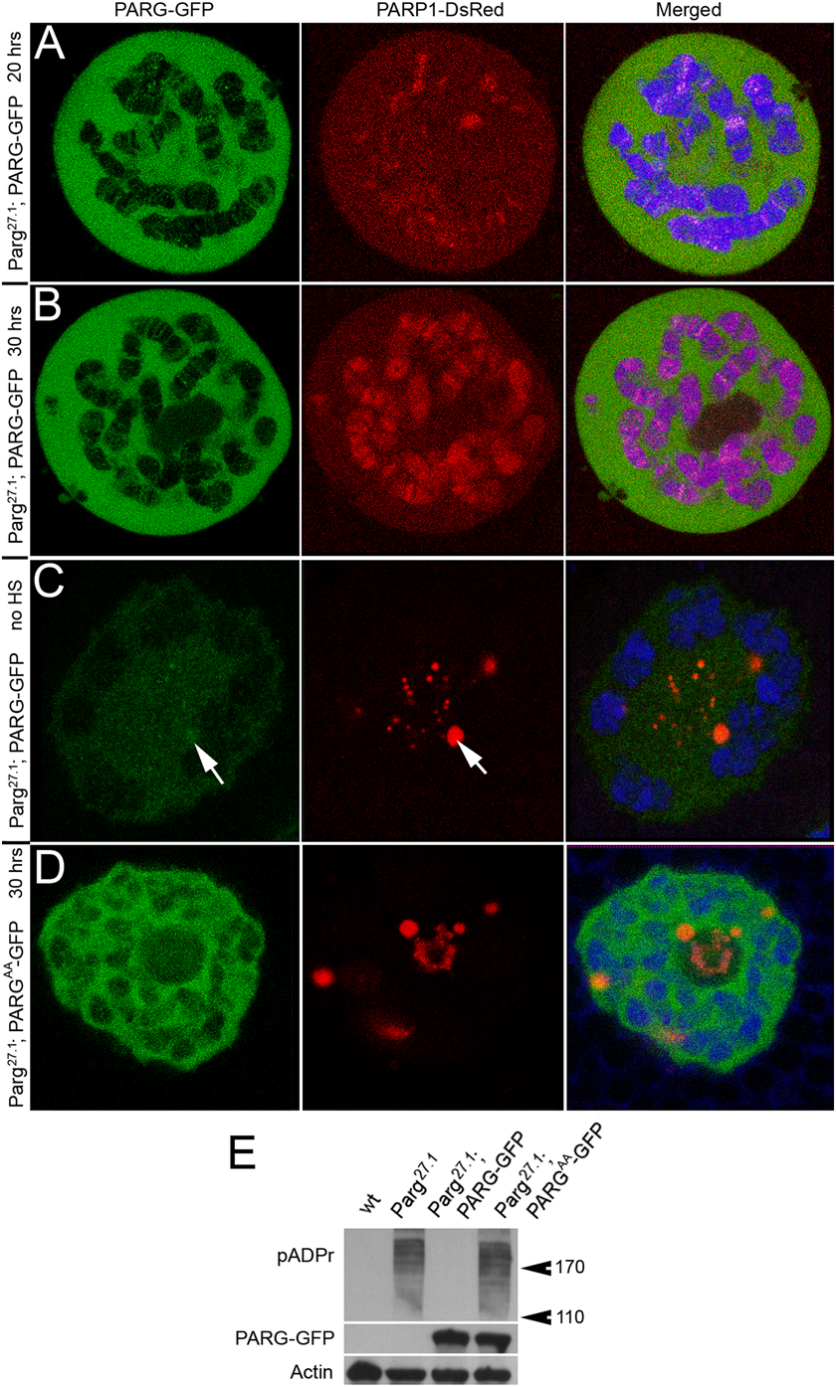
The ectopic expression of recombinant PARG-EGFP protein in *Parg^27.1^* mutant animals rescues PARP1-DsRed localization to chromatin. The expression of UAS::PARG-EGFP transgene was induced in early second-instar larvae (A, B), or in early third-instar larvae (C), using hsp::Gal4 driver induction. Salivary glands were dissected and analyzed by confocal microscopy after 20 hrs following Gal4 induction (A, C) and after 30 hrs following Gal4 induction (B). PARG-EGFP protein is green; PARP1-DsRed is red, and DNA (Draq5) is blue. An arrow indicates colocalization of PARG-EGFP and PARP1-DsRed in the CB-like particle. D. The expression of recombinant catalytically inactive PARG^AA^-EGFP protein in *Parg^27.1^* mutant animals does not rescue PARP1-DsRed protein localization to chromatin (after 30 hrs following Gal4 induction). E. Western blot hybridization was used to detect the accumulation of pADPr in *Parg^27.1^* mutant and *Parg^27.1^* mutant expressing PARG^AA^-EGFP protein, but not in wild-type and *Parg^27.1^* mutant expressing PARG-EGFP protein.

In the following experiments, we examined if inducible expression of recombinant PARG protein could suppress accumulation of PARP1-DsRed protein in HLB/CBs and rescue the *parg^27.1^* mutant phenotypes. To accomplish this, we expressed a previously constructed epitope-tagged version of UASt::PARG-EGFP transgenic construct [10] in *parg^27.1^* mutant animals, along with PARP1-DsRed. When expressed using constitutive 69B Gal4-driver [25], PARG-EGFP rescues *parg^27.1^* lethality. Using temperature-inducible hsp::Gal4 activator (gift of G. Cavalli lab), we were also able to suppress *parg^27.1^* phenotypes. When expression of hsp::Gal4 was induced before third-instar larvae stage, the PARG-EGFP protein was strongly accumulated in nucleoplasm (Figure 5A, B). No ectopic PARP1-DsRed-containing bodies were observed in those animals. Moreover, 10–20 hours after Gal4 transgene induction, the PARP1-DsRed protein accumulation appeared in a subset of chromosomal loci (Figure 5A); after 30 hours, the PARP1-DsRed protein distribution in chromatin was similar to that of the wild-type (Figure 5B). Late expression of the PARG-EGFP (in third-instar larvae) did not rescue *parg^27.1^* mutants. In this case, the accumulation of PARG-EGFP in nucleoplasm still occurs, but the amount of PARG protein is not sufficient to rescue mutant phenotypes. Therefore, the nucleoplasmic bodies persist and absorb the PARP1-DsRed (Figure 5C). It is also interesting to note that such conditions result in the presence of a detectable amount of PARG-EGFP protein in the HLB/CBs (Figure 5C). This last result suggests that the PARG protein, as well as PARP1, may be one of the HLB/CB components. Moreover, the same experiment performed with catalytically inactive PARG^AA^-EGFP proteins (see Material and Methods) does not rescue PARP1 protein localization to chromatin (Figure 5D) and does not suppress accumulation of pADPr (Figure 5E). This, in turn, implicates that the regulation of pADPr turnover may be one function of HLB/CB. If this is true, then poly(ADP-ribosyl)ated proteins necessarily pass through CB, giving further evidence that PARP1 is a key component of the CB transcription machinery, as proposed above. Considered together, these results 1) support the hypothesis that PARG enzyme cleaves pADPr in HLB/CBs and recycles proteins modified in chromatin and nucleoli, including PARP1 protein itself and 2) implicate these processes in maintaining control over the protein flow through Cajal bodies, a dynamic that we have proposed and demonstrated in this report.

### Protein Components of HLB/CBs Are Targets for Poly(ADP-ribosyl)ation Reaction

**Figure 6:**
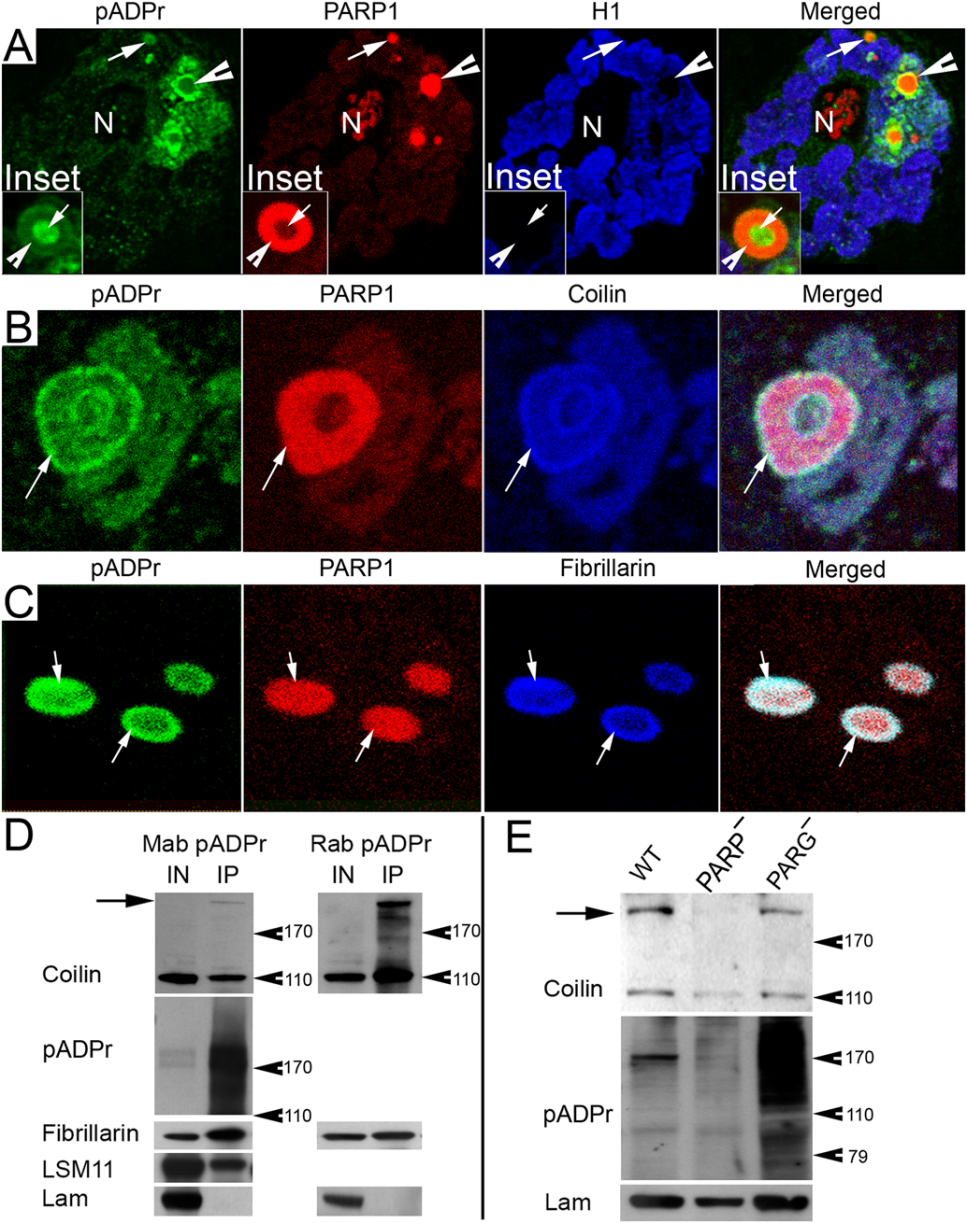
The components of Cajal body are targets for pADPr. (A) The immunostaining of thick sections prepared from *Parg^27.1^* mutant salivary gland is shown. “Free” nucleoplasmic bodies (arrow) and “chromatin-embedded” CBs (arrowhead) are shown. Specific antibodies were used: rabbit anti-pADPr (green) and mouse anti-histone H1 (blue). PARP1-DsRed (red) was visualized by DsRed autofluorescence. Inset. The single CB-like particle is magnified. Arrow indicates accumulation of pADPr in the CB cavity. Arrowhead shows enrichment of PARP1 protein in the CB matrix. (B, C) Immunostaining of *Parg^27.1^* mutant salivary gland is shown. Specific antibodies were used: mouse (10H) anti-pADPr (green) (B–C); Guinea Pig anti-Coilin (blue) (B) and rabbit anti-Fibrillarin (C). PARP1-DsRed (red) was visualized by DsRed autofluorescence. (B) The single chromatin-embedded CB-like particle is presented. Arrow indicates the colocalization of Coilin and pADPr on periphery of CB. (C) Three CBs are shown. Arrows show the colocalization of Fibrillarin and pADPr on periphery of CBs. (D) Immunoprecipitation assays using mouse and rabbit antibody against pADPr. Wild-type *Drosophila* stock was used to prepare protein extracts. To detect protein on Western blots, the following antibodies were used: Guinea Pig anti-Coilin, mouse anti-pADPr, rabbit anti-Fibrillarin, rabbit anti-LSM11 and mouse anti-Lamin C. Arrow indicates hypermodified isoform of Coilin. (E) Western blot analysis of total protein extracts from wild-type (WT), *Parp1* (PARP^−^) and *Parg* (PARG^−^) mutant flies was performed. Rabbit anti-Coilin antibody was used. Arrow indicates hypermodified isoform of Coilin.

Multiple *in vivo* and *in vitro* observations (reviewed in [2]) report that the main, if not the exclusive, target for pADPr reaction is the PARP1 protein itself. Therefore, we investigated whether most pADPr molecules co-migrate into the same nucleoplasmic domains where PARP1 is localized. In order to test this in respect to the ultrastructure of ectopic HLB/CBs, we performed immunostaining of thick sections prepared from the salivary glands of *parg^27.1^* mutant third-instar larvae. As the PARP1-DsRed protein relocated from chromatin, we observed two types of nucleoplasmic PARP1-containing particles: “embedded” in chromatin (one or four per nucleus) (Figure 6A; Figure S7, arrowhead) and “free” nucleoplasmic (five to forty per nucleus) (Figure 6A; Figure S7, arrow). The position of “embedded” bodies is similar to that of normal CBs [22], and these “embedded” bodies also often demonstrate structural properties similar to those of classical CBs [20] by showing PARP1-positive CB matrix and pADPr-positive CB cavity (Figure 6A, Inset; Fig S7A, Inset). As expected [10], a significant amount of pADPr is associated with these PARP1-containing bodies (Figure 6A). However, the “chromatin-embedded” particles demonstrated separation of PARP1-DsRed from the main pool of pADPr (Figure 6A, Inset). To confirm this segregation, we performed immuno-gold staining of thin sections prepared from the same materials, followed by electron microscopy.

“Free” nucleoplasmic bodies always demonstrate strong staining for pADPr (Figure S7B) and PARP1 (Figure S7C). “Chromatin-embedded” bodies are also positive for pADPr and PARP1, but they are surrounded with material which contains significant amounts of pADPr, but no PARP1 (Figure S7E–F). These results demonstrate that a significant portion of pADPr accumulates separately from PARP1 protein. Therefore, we tested protein components of CB for modification with pADPr. We found that both Coilin and Fibrillarin always completely colocalized with pADPr in HLB/CB (Figure 6 B–C). This suggests that those proteins are bound to pADPr. To confirm this, we performed co-immunoprecipitation experiments using antibodies against pADPr. We used total protein extracts from wild-type flies. We found that Coilin, as well as Fibrillarin, always co-purify with pADPr (Figure 6D). Moreover, Western blots revealed the existence of a heavy isoform of Coilin protein, which specifically co-purified with anti-pADPr antibody and disappeared in protein extracts from *PARP1* mutant nuclei (Figure 6E). An additional co-immunoprecipitation experiment using protein extracts from *Drosophila* cell culture also confirmed direct interaction of CB and HLB components with pADPr (Figure S8). These data allow us to hypothesize that Coilin, Fibrillarin and LSM11 are targets for enzymatic activity of PARP1 protein and pADPr-interacting proteins.

## Discussion

Immunofluorescence studies of mammalian and *Drosophila* nuclei indicate that PARP1 has widespread localization in chromatin [4],[5],[8],[26],[27]. Considerable evidence suggests that PARP1 is a regular constituent of chromatin [28],[29]. The PARP1 protein is required for chromatin assembly, maintenance of an initially silent state of transcription, and transcriptional activation [6],[8],[30]. As demonstrated by the results enumerated in this paper, 1) PARP1 regulates protein localization to Cajal body and 2) PARP1 automodification plays a further key regulatory role in this activity. Specifically, PARP1 activation, followed by automodification by PARP1 molecules, is critical for PARP1 protein removal from chromatin [9] and subsequent localizing to CB. To accomplish this, PARP1 protein is shuttled between chromatin and CBs. In the *Parg* mutants, most PARP1 protein is automodified, and the balance is shifted toward accumulation of PARP1 in CBs [10]. Beyond protein localization into CB, however, we have further addressed the significance of protein interaction with pADPr *in vivo* in relation to CB integrity. In this paper, we have characterized this phenomenon by testing the mediation of the interaction between the key protein Coilin and other components of CB, such as Fibrillarin. Therefore, since interaction of Coilin and Fibrillarin can be disrupted in PARP1 mutants and since CB has been shown to disintegrate into multiple small particles, we have also demonstrated that PARP1 is crucial for maintaining CB integrity. Thus, we hypothesize that it is the PARP1/PARG protein machinery which provides the mechanism that controls the shuttling of protein complexes between nucleolus and chromatin and CBs.

### Automodified PARP1 Is a Shuttle for Protein Delivery to Cajal Body

**Figure 7:**
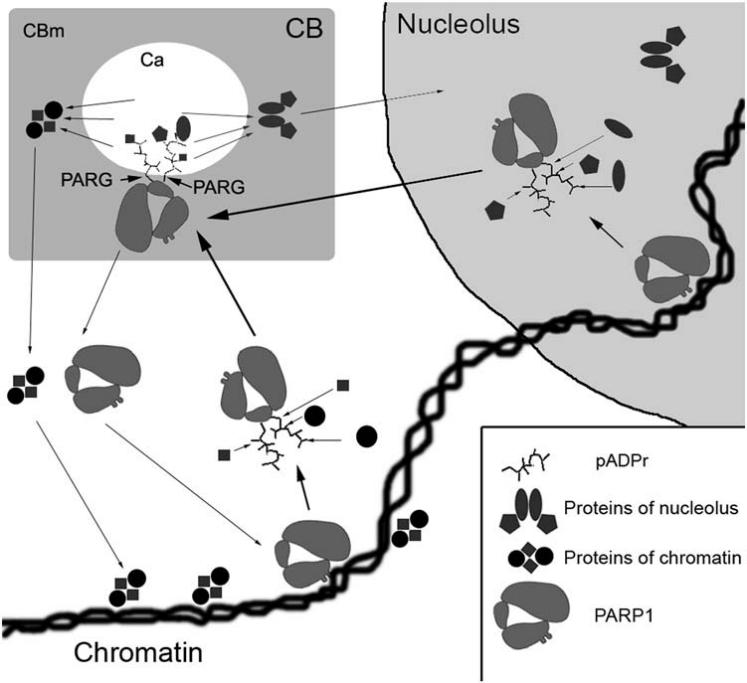
A model of protein delivery to Cajal body by PARP1 shuttling is shown. (1) PARP1 protein is localized in chromatin and nucleoli. (2) Upon activation, PARP1 automodifies and (3) gains the ability to bind by pADPr a number of proteins with pADPr-binding domain. (4) Whole complex consisting of automodified PARP1 and proteins seated on pADPr migrates into Cajal bodies. (5) In CB, complex is disassembled as a result of cleavage of pADPr and released proteins are recycled. PARP1, pADPr protein-complexes of chromatin and nucleolus are indicated. CBm = Cajal body matrix, and Ca = Cajal body cavity.

Most detected *in vivo* acceptors of pADPr are chromatin proteins (see [2]), but the significance of this modification *in vivo* is unclear. Since 90–95% of total pADPr is covalently bound to PARP1 itself, the remaining 5–10% of modification by pADPr could be considered as background. Beyond this possibility, however, the dramatic difference between levels of incorporation of ADPr to PARP1 and to other proteins allows us to hypothesize that acceptors of pADPr are not in and of themselves important. Rather, it is the generation of huge, charged shells of pADPr alone that may cause local changes of nuclear architecture and chromatin structure [8],[31],[32]. To explain, large branches of pADPr (i.e., formal negative charge of phosphate groups) may specifically “attract” a number of chromatin proteins and thus cause local temporal removal of those components from chromatin. This specific ability to bind pADPr has been reported for a large list of proteins [33],[34],[35],[36]. In fact, a number of papers report that chromatin changes caused by PARP1 are reversible by degradation of pADPr by PARG [37],[38] and that the same proteins are then re-utilized for chromatin reassembly. These observations finally led to the model of “histone shuttling” during PARP-induced chromatin changes [31],[32],[39]. However, since the present study reports the accumulation of automodified PARP1 protein, proteins modified by PARP1 and proteins interacting with pADPr in Cajal bodies (CBs), we propose a model whereby automodified PARP1 may directly deliver components of chromatin and nucleolus to CBs in a way that permits their release by PARG, thus allowing their reassembly into active protein complexes (Figure 7).

### Cajal Bodies: An Organelle for Protein Recycling?

Cajal bodies (CBs) are small nuclear organelles detected in the nuclei of many eukaryotes [40]. Many components of CBs are shared with the nucleolus, and CBs frequently localize to the nucleolar periphery or within the nucleoli [41]. Although their function is not fully understood, recent work suggests that they are involved in several nuclear functions, including modification of small nuclear RNAs and small nuclear ribonucleoproteins. Such modification is important for the assembly of the eukaryotic RNA polymerases that are then transported as multiprotein complexes to specific chromatin domains [41]. CBs have been implicated in replication-dependent histone gene transcription and mRNA maturation [42],[43], and a subset of CBs is physically associated with histone gene clusters (HLB) [44]. Therefore, up to now, a general model of CB function has been represented by preassembly of nuclear protein complexes for their performance. We have, however, suggested that inactivated automodified PARP1 proteins accumulate in CB which leads to the construct of a different model for CBs in the nuclei. Specifically, most previous reports proposed initial (post-synthesis) translocation of a given protein into CBs, followed by modification, assembly and transportation of matured components toward a place of their final destination and work [21]. In the case of the *parg^27.1^* mutant, however, we detected an opposite order of events with the PARP1 protein. That is, PARP1 is normally localized and active in chromatin [6]. Each cycle of PARP1 protein activation then terminates by automodification [9],[10]. Based on our findings, however, PARP1 next relocates from chromatin into CB after its automodification and is subsequently inactivated. In the case of PARP1 protein, at least, because CB is important for recovery of inactive hypermodified protein, this sequence of events necessarily leads to the suggestion that CB is an organelle for the disassembly and recovery of protein complexes after their work has been done and, thus, supports a novel recently suggested paradigm of CB function [45].

In summary, we have demonstrated that poly(ADP-ribosyl)ation is required for protein delivery into CB. Furthermore, interaction of the key protein Coilin with other protein components of CB, such as Fibrillarin, depends upon the activity of PARP1 and PARG proteins. These results provide the first molecular explanation for the roles that PARP1/PARG protein machinery play in the regulation of nucleolar organelle biogenesis and dynamics. Finally, our findings suggest the novel role of CB in nuclear protein recycling and emphasize the importance of increasing our knowledge about the properties of poly(ADP-ribose) dynamics *in vivo*.

## Materials and Methods

### *Drosophila* Strains and Genetics

Genetic markers are described in FlyBase [46], and stocks were obtained from the Bloomington Stock Center, except as indicated. *Parg^27.1^* was constructed by Hanai et al. [16]. pP{w1, UAST::PARP1-DsRed}, called UAS::Parp1-DsRed, was described in Tulin et al., 2002 [6]. The transgenic stock with pP{w1, UAST::PARG-EGFP}, called UAS::Parg-EGFP, was described in Tulin et al. [10]. The following GAL4 driver strains were used: da::GAL4 (gift of A. Veraksa), 69B-GAL4 [25], hs::GAL4 (gift of G. Cavalli lab), and arm::GAL4 (Bloomington stock no. 1560). To induce expression from the hs::GAL4 driver, *Drosophila* larvae were heat-shocked for 1 hr at 37°C twice daily for 2 days prior to the second-instar stage. Balancer chromosome carrying Kr::GFP, i.e., FM7i, P{w1, Kr-GFP} [47], was used to identify heterozygous and homozygous *Parg^27.1^*.

### Construction of Transgenic *Drosophila*

To construct UAS::PARP1-ECFP, we generated full-length PARP1 ORF using PCR. Primers were used: Parp1d, CACCatgtctgattctgcagttg, and Parp1r, ctttttggcagccgtag. We used wild-type *Drosophila* total cDNA as a template for PCR. The resulting PCR products were cloned through The Drosophila Gateway™ Vector Cloning System (Carnegie Institution of Washington) into a corresponding vector for *Drosophila* transformation. To make a catalytically inactive PARG-EGFP transgenic construct, two evolutionarily conserved [48],[49] glutamic acid residues (at 385 and 386 positions, according to *Drosophila* PARG sequence) were mutated to alanines, generating UAS::Parg^AA^-EGFP transgenic construct. Transformation was as described in [50], with modifications [51].

### Immunoprecipitation

Lysates for immunoprecipitation were prepared as follows: 15 third-instar larvae were collected for each sample. They were put into Eppendorf tubes and rinsed 3 times with 1 ml of dist. water. 500 ul of ice-cold lysis buffer (10 mM Tris-HCl pH 7.5, 150 mM NaCl, 1 mM EDTA, 0.2% NP40, 1% Triton ×100, 0.1% SDS, 1% Sodium Deoxycholate, complete TM protease inhibitors (Roche) and 0.1 mM Pefabloc SC (Fluka)) were added to each tube, and larvae were homogenized by hand pestle homogenizer on ice. After 30 min incubation on ice, samples were centrifuged at 14500 RPM for 20 min (4°C). Supernatants were transferred to new Eppendorf tubes on ice. For one immunoprecipitation reaction, 500 mkl of total lysates were incubated with 25 mkl Protein-G Sepharose 4B (Sigma #P3296-5ML) on a rotating platform for 1 hr 30 min at 4°C. Beads were removed by spinning 1 min, 2000 g. Appropriate amount of antibody was added to the lysates, and the mixture was incubated 4 hrs on a rotating platform at 4°C. The following antibodies were used for immunoprecipitation: anti-Coilin (Guinea Pig, 1∶4000, J. Gall) [52], anti-Coilin (Rabbit, 1∶4000, J. Gall) [52], anti-pADPr (Rabbit, 1∶50, Calbiochem, #528815), anti-pADPr (Mouse monoclonal, 1∶20, Tulip, #1020), anti-GFP (Rabbit, 1∶100, Torrey Pines, #TP401). Then, 30 mkl of Protein-G Sepharose 4B were added to the lysates and incubated overnight at 4°C with rotation. Beads were washed 4 times for 5 min in 1.0 ml of the lysis buffer. Bound proteins were eluted by 60 mkl of 1× Laemli with heating at 95°C for 5 min.

### Electron Microscopy

For ultrastructural analysis, the salivary glands were dissected, fixed in 2% formaldehyde/ 2% glutaraldehyde in 0.1 M cacodylate buffer pH 7.2, post-fixed in 1% OsO_4_, dehydrated in ethanol and propylenoxide, and embedded in EMbed-812 (EMS, Fort Washington, PA) in flat molds. After polymerization for 60 hours at 65°C, 70 nm sections were cut on Leica Ultracut E microtome (Leica, Austria), placed on collodion/carbon-coated grids, and stained with 2% uranyl acetate/ lead citrate. Sections were viewed on a Tecnai 12 transmission electron microscope (TEM) (FEI, Hillsboro, OR).

For EM immunocytochemistry, samples were prepared according to Tokuyasu 1980 [53]. In brief, the dissected salivary glands were fixed in 4% formaldehyde/0.2% glutaraldehyde in 0.1 M PHEM (60 mM PIPES, 25 mM HEPES, 2 mM MgCl_2_, 10 mM EGTA, pH 6.9), cryo-protected in 2.3 M sucrose, mounted on aluminium pins, and frozen in liquid nitrogen. Thin frozen sections were then cut on a Leica EM UC6/FC6 cryomicrotome (Leica, Austria), collected on a drop of sucrose/methylcellulose mixture and placed on a formvar-carbon grid. The sections were labeled with primary antibody, and the label was subsequently visualized by colloidal gold conjugated to Protein A. Sections were stained/embedded in 2% methylcellulose/0.2% uranyl acetate and observed under a Tecnai 12 TEM.

### Immunostaining of Thick Sections

For immunofluorescence, frozen samples prepared for TEM were cut to 0.5 µm-thick sections and labeled with primary antibodies followed by secondary antibodies conjugated to fluorochromes. Sections were washed in 200 mkl drops of 1XPBS+0.1% glycin solution 5 times for 1 min (Coverslip was placed face down on a drop), then blocked in 1XPBS+1%BSA twice for 3 min and incubated with primary antibody dissolved in blocking solution in humid chamber for 1 h. Afterwards, sections were washed with 1XPBS (4 times for 2 min) and incubated with secondary antibody in a humid chamber for 40 min. The mount was then washed with 1XPBS (3 times for 5 sec and 4 times for 2 min) and mounted in Vectashield (Vector Laboratories, Burlingame, CA). Samples were examined by confocal microscopy using a Leica TCS-LT microscope. The primary antibodies used were as follows: mouse monoclonal (mAb) H1 (Santa Cruz Biotechnology, sc-8030) (1∶400 or 1∶1000), and pAb PAR (Calbiochem) (1∶5000). Mouse Alexa-488, rabbit Alexa-568 and rabbit Alexa-633 (Molecular Probes) were used as secondary antibodies (1∶400). For DNA staining, propidium iodide was used at 0.05 mg/ml final concentration in Vectashield or Draq5™ (#BOS-889-001, Alexis Biochemicals), and up to 2.5 µM was added to the final wash with 1XPBS for 2 min before mounting in Vectashield.

### Fluorescent In Situ Hybridization (FISH)

Fluorescent U85 scaRNA probe labeled with Alexa-488-UTP was prepared by *in vitro* transcription reaction from genomic PCR amplified product as described previously [22],[23]. For amplification of U85 scaRNA genomic fragment, the following primers were used: 5′ TAGGGCGGAATTCATTAACCCTCACTAAAGGGATGCCCATGATGAAATATTCGAC 3′ TAGGGCTCTAGATAATACGACTCACTATAGGGATCCTTGCGCTCAGATTACTAAAGACG.

Since this RNA probe was generated using polymerase T3 transcription, T7 polymerase was used for antisense. Probes were diluted in hybridization solution (50% formamide, 5XSSC, 10 mM citric acid, 50 ug/ml heparin, 500 ug/ml yeast tRNA, 0.1% Tween20). Hybridization was performed overnight at 42°C.

## Supporting Information

Figure S1 Small PARP1- and pADPr-positive extrachromosomal particles detected by confocal microscopy in *Drosophila* nuclei. The size and localization of these particles suggest certain similarities to Cajal bodies (CBs) and the related organelle, histone locus body (HLB). A. The dissected salivary glands expressing PARP1-DsRed (red) transgenic construct were stained with the DNA binding dye Draq5 (green). Position of extrachromosomal particles is indicated with arrows. N - nucleolus. B. The dissected salivary glands from wild-type *Drosophila* were fixed and partially squashed on a slide, followed by immunostaining with anti-pADPr antibody (red) and with the DNA binding dye DAPI (blue). Position of extrachromosomal particles is indicated with arrowheads. N - nucleolus.
(3.62 MB TIF)

Figure S2 Control experiments confirm the specificity of PARP protein interaction with proteins of Cajal body. A. Fibrillarin interacts with PARP protein in vivo. Co-immunoprecipitation assays using rabbit anti-Fibrillarin antibody. Wild-type (WT) *Drosophila* stock (negative control) and stock expressing PARPe-EGFP were used. To detect PARPe-EGFP protein on Western blots, mouse monoclonal anti-GFP antibody was used. B. Nuclear GFP protein does not interact with Coilin. Co-immunoprecipitation assays using rabbit anti-GFP antibody. *Drosophila* stock expressing Nuclear-GFP protein was used. To detect proteins on Western blots, the following antibodies were used: mouse anti-GFP; Guinea Pig anti-Coilin and mouse anti-Actin. C. Preimmune serums from rabbit (Rab IgG), Guinea Pig (GP IgG) and mouse (Mab IgG) do not cross react with components of Cajal and Histone locus bodies. Co-immunoprecipitation assays using preimmune serums. Wild-type (WT) *Drosophila* stock and stock expressing PARPe-EGFP were used. To detect proteins on Western blots, the following antibodies were used: mouse anti-GFP; Guinea Pig anti-Coilin, rabbit anti-Fibrillarin, rabbit anti-LSM11 and mouse anti-Lamin C. D. Intrinsic PARP protein interacts with coilin. Immunoprecipitation assays using rabbit anti-PARP1 and anti-GFP antibodies. Wild-type *Drosophila* stock was used. To detect proteins on Western blots, the following antibodies were used: Guinea Pig anti-Coilin and mouse anti-Actin.
(0.35 MB TIF)

Figure S3 PARP1 gene and PARP1-DsRed transgene expression during *Drosophila* development in wild-type (wt) and *Parg27.1* mutant animals. Northern blot hybridization using DsRed (to detect transgenic construct expression), PARP1 (total PARP1 RNA) and Tubulin (loading control) DNA probes.
(0.24 MB TIF)

Figure S4 The inhibition of PARG function induces ectopic CBs. (A–C) The electron micrographs of wild-type (A) and *Parg27.1* mutant (B) salivary gland nucleus and diploid *Parg27.1* mutant nucleus (C) are shown. The arrows indicate nucleoplasmic bodies which are specific for the *Parg27.1* mutant.
(1.75 MB TIF)

Figure S5 RNA Polymerase II subunits are localized into ectopic CB in *Parg27.1* mutant nuclei. Immuno-gold staining of ectopic CB particles using (A) antibodies against Pol II CTD domain and (B) anti-rpb4 subunit antibody. Specific localization of immuno-gold staining (black dots) in CB is clearly demonstrated.
(1.09 MB TIF)

Figure S6 Ecdysteroid hormone controls PARP1 protein localization in vitro. Salivary glands from wild-type (A) and *Parg27.1* mutant (B, C), third-instar larvae, expressing PARP1-DsRed (red), were cultured in the presence (A, B) or absence (C) of ecdysteroid hormone (20E), followed by confocal microscopy. DNA was visualized with Draq5 staining (green). Nucleoplasmic PARP-containing bodies induced in *Parg27.1* mutant nuclei (B) are indicated with an arrow. N - nucleolus.
(3.26 MB TIF)

Figure S7 The inhibition of PARG function induces ectopic CBs and relocation of PARP1 protein from chromatin into CBs. (A) The electron micrograph of *Parg27.1* mutant nucleus is shown. The “free” nucleoplasmic (arrow) and “chromatin-embedded” (arrowhead) particles are shown. Inset. The cross section through the single CB-like particle is magnified (CBm - Cajal body matrix; Ca - cavity). (B–D) Immuno-gold staining of “free” nucleoplasmic particles using antibodies (1) against pADPr (B), (2) against DsRed (C) and (3) against H1 (D) is shown. (E–G) Immuno-gold staining of “chromatin-embedded” particles using antibodies (1) against pADPr (E), (2) against DsRed (F) and (3) against H1 (G) is shown. Arrowhead indicates CB itself; arrow indicates surrounding materials accumulating on pADPr (E), but not PARP1 (F).
(3.98 MB TIF)

Figure S8 Key proteins of Cajal body and Histone locus body interact with pADPr in *Drosophila* S2 cells. Co-immunoprecipitation assays using mouse antibody against pADPr (10H). S2 cell culture was used to prepare protein extracts. To detect protein on Western blots, the following antibodies were used: Guinea Pig anti-Coilin, rabbit anti-Fibrillarin, rabbit anti-LSM11 and mouse anti-Lamin C.
(0.17 MB TIF)
