# Supplementary figures and images for: Poly (ADP-Ribose) Polymerase 1 Is Required for Protein Localization to Cajal Body
Source: PLoS Genet. 2009 Feb 20;5(2):e1000387. doi: 10.1371/journal.pgen.1000387 (PMC2637609; doi:10.1371/journal.pgen.1000387)

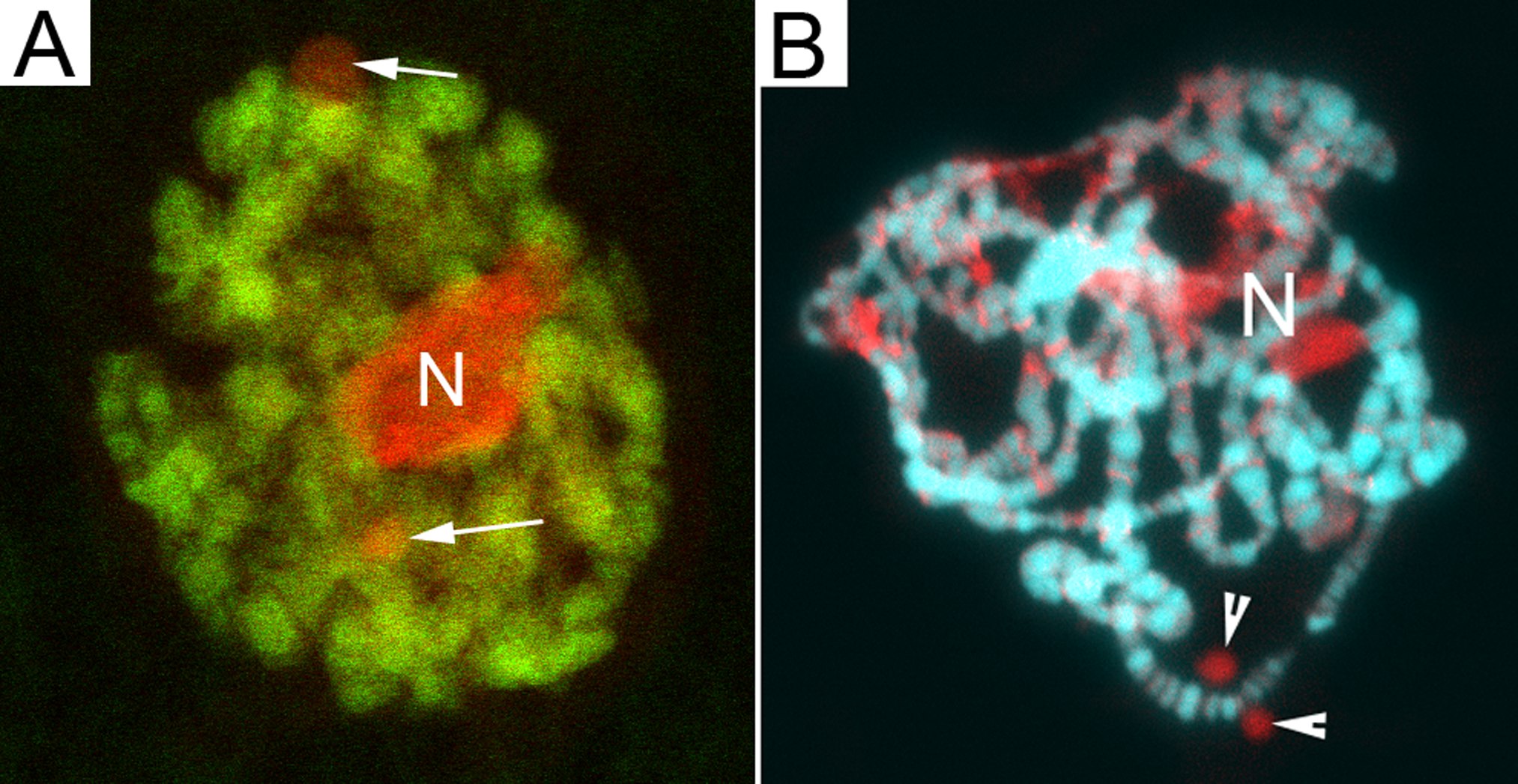

Supplement: Figure S1 — Small PARP1- and pADPr-positive extrachromosomal particles detected by confocal microscopy in Drosophila nuclei. The size and localization of these particles suggest certain similarities to Cajal bodies (CBs) and the related organelle, histone locus body (HLB). A. The dissected salivary glands expressing PARP1-DsRed (red) transgenic construct were stained with the DNA binding dye Draq5 (green). Position of extrachromosomal particles is indicated with arrows. N - nucleolus. B. The dissected salivary glands from wild-type Drosophila were fixed and partially squashed on a slide, followed by immunostaining with anti-pADPr antibody (red) and with the DNA binding dye DAPI (blue). Position of extrachromosomal particles is indicated with arrowheads. N - nucleolus. (3.62 MB TIF) [file pgen.1000387.s001.tif]

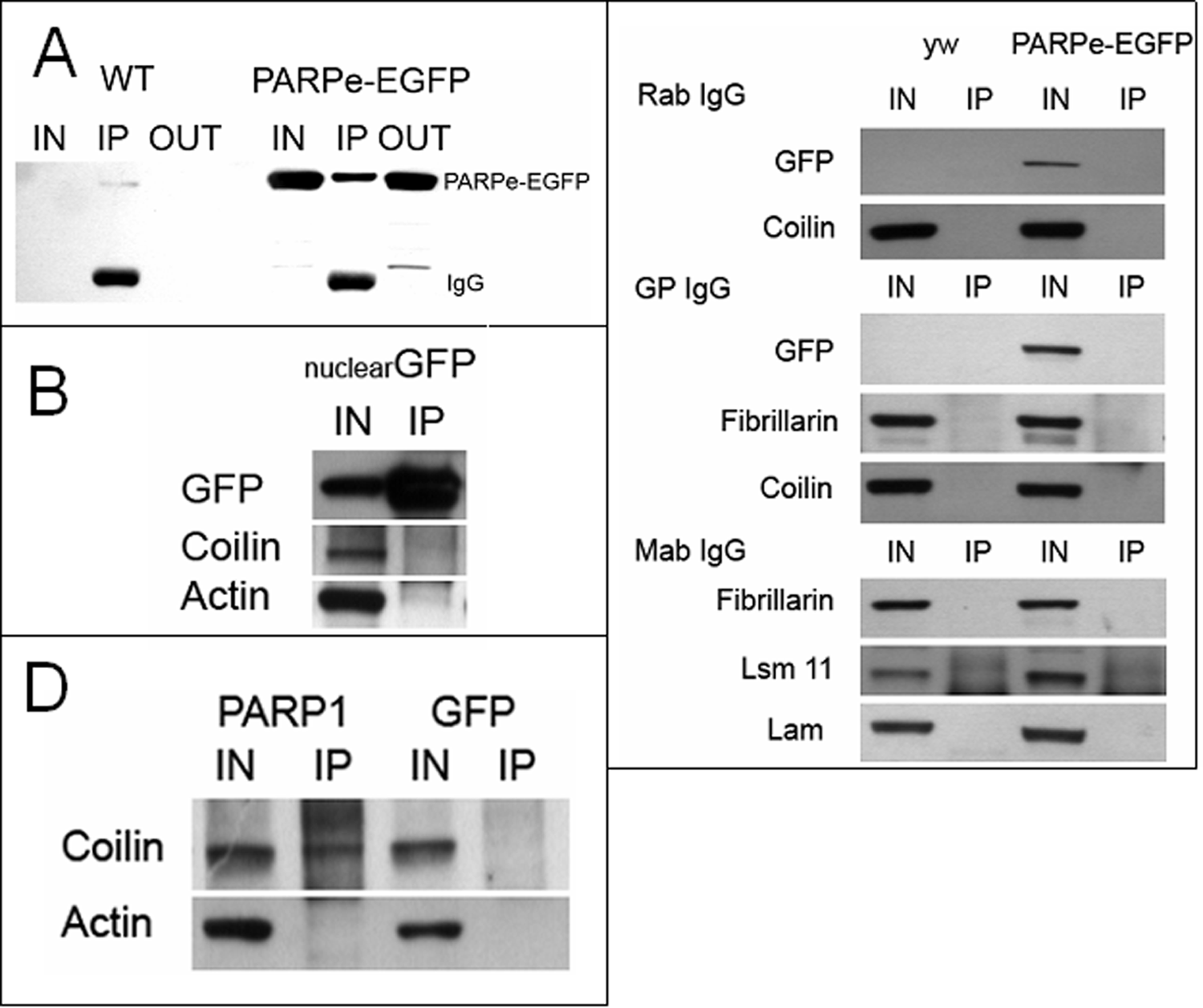

Supplement: Figure S2 — Control experiments confirm the specificity of PARP protein interaction with proteins of Cajal body. A. Fibrillarin interacts with PARP protein in vivo. Co-immunoprecipitation assays using rabbit anti-Fibrillarin antibody. Wild-type (WT) Drosophila stock (negative control) and stock expressing PARPe-EGFP were used. To detect PARPe-EGFP protein on Western blots, mouse monoclonal anti-GFP antibody was used. B. Nuclear GFP protein does not interact with Coilin. Co-immunoprecipitation assays using rabbit anti-GFP antibody. Drosophila stock expressing Nuclear-GFP protein was used. To detect proteins on Western blots, the following antibodies were used: mouse anti-GFP; Guinea Pig anti-Coilin and mouse anti-Actin. C. Preimmune serums from rabbit (Rab IgG), Guinea Pig (GP IgG) and mouse (Mab IgG) do not cross react with components of Cajal and Histone locus bodies. Co-immunoprecipitation assays using preimmune serums. Wild-type (WT) Drosophila stock and stock expressing PARPe-EGFP were used. To detect proteins on Western blots, the following antibodies were used: mouse anti-GFP; Guinea Pig anti-Coilin, rabbit anti-Fibrillarin, rabbit anti-LSM11 and mouse anti-Lamin C. D. Intrinsic PARP protein interacts with coilin. Immunoprecipitation assays using rabbit anti-PARP1 and anti-GFP antibodies. Wild-type Drosophila stock was used. To detect proteins on Western blots, the following antibodies were used: Guinea Pig anti-Coilin and mouse anti-Actin. (0.35 MB TIF) [file pgen.1000387.s002.tif]

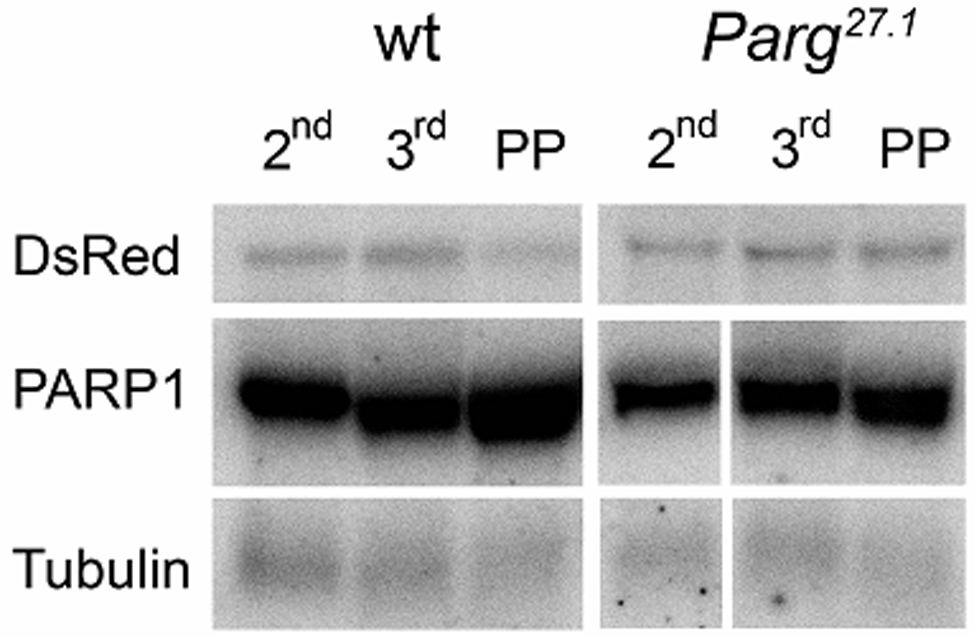

Supplement: Figure S3 — PARP1 gene and PARP1-DsRed transgene expression during Drosophila development in wild-type (wt) and Parg27.1 mutant animals. Northern blot hybridization using DsRed (to detect transgenic construct expression), PARP1 (total PARP1 RNA) and Tubulin (loading control) DNA probes. (0.24 MB TIF) [file pgen.1000387.s003.tif]

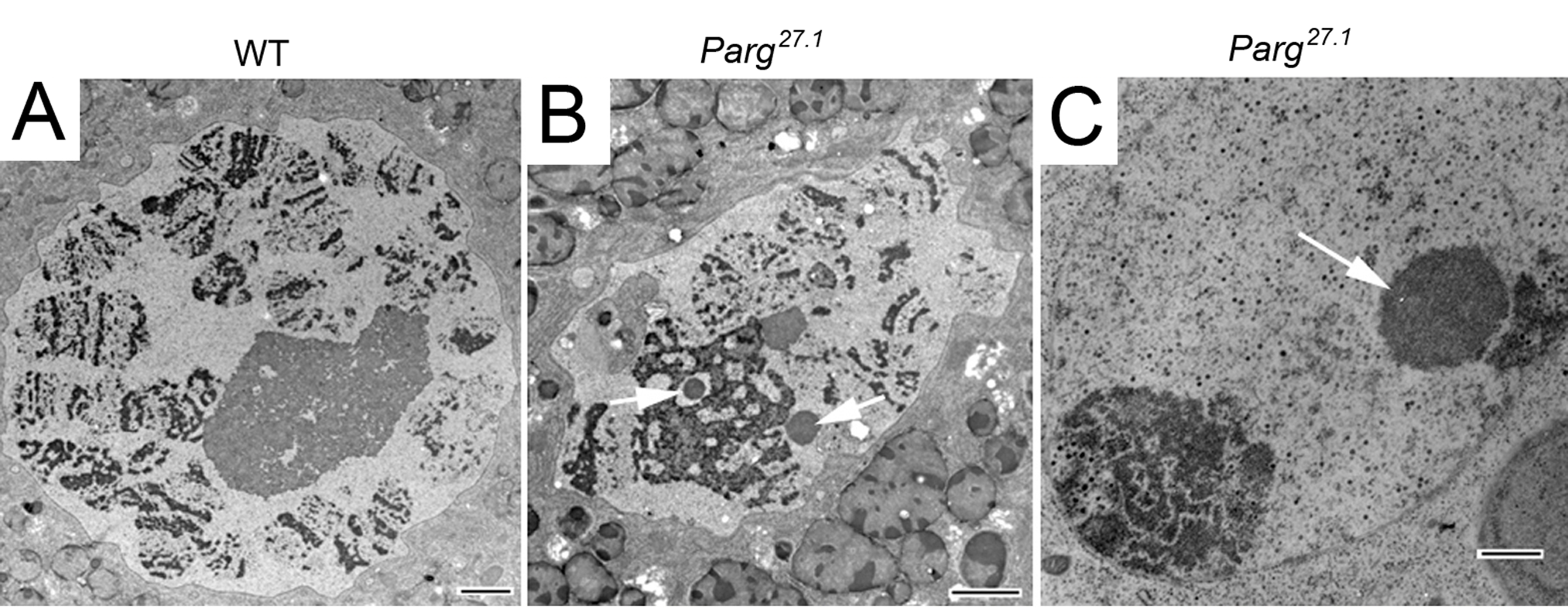

Supplement: Figure S4 — The inhibition of PARG function induces ectopic CBs. (A–C) The electron micrographs of wild-type (A) and Parg27.1 mutant (B) salivary gland nucleus and diploid Parg27.1 mutant nucleus (C) are shown. The arrows indicate nucleoplasmic bodies which are specific for the Parg27.1 mutant. (1.75 MB TIF) [file pgen.1000387.s004.tif]

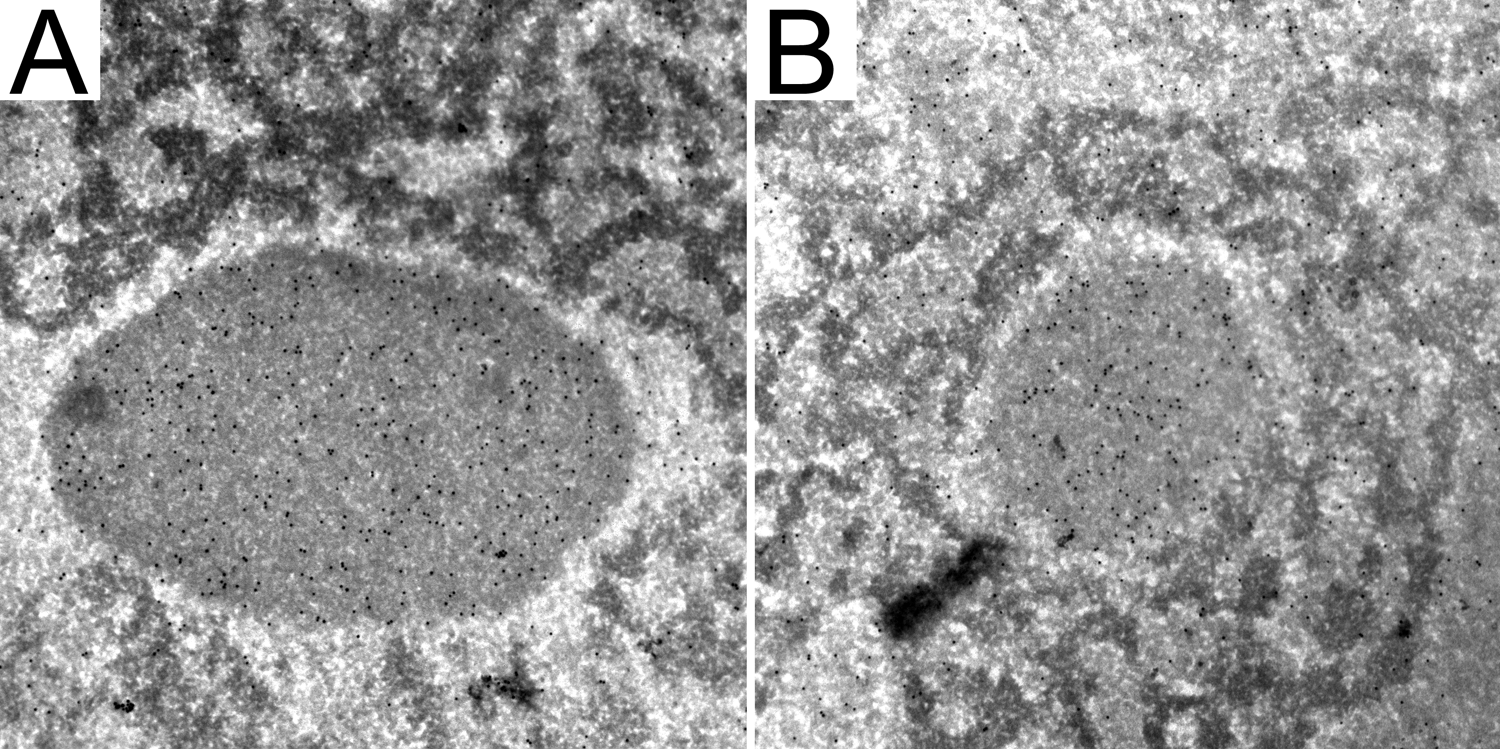

Supplement: Figure S5 — RNA Polymerase II subunits are localized into ectopic CB in Parg27.1 mutant nuclei. Immuno-gold staining of ectopic CB particles using (A) antibodies against Pol II CTD domain and (B) anti-rpb4 subunit antibody. Specific localization of immuno-gold staining (black dots) in CB is clearly demonstrated. (1.09 MB TIF) [file pgen.1000387.s005.tif]

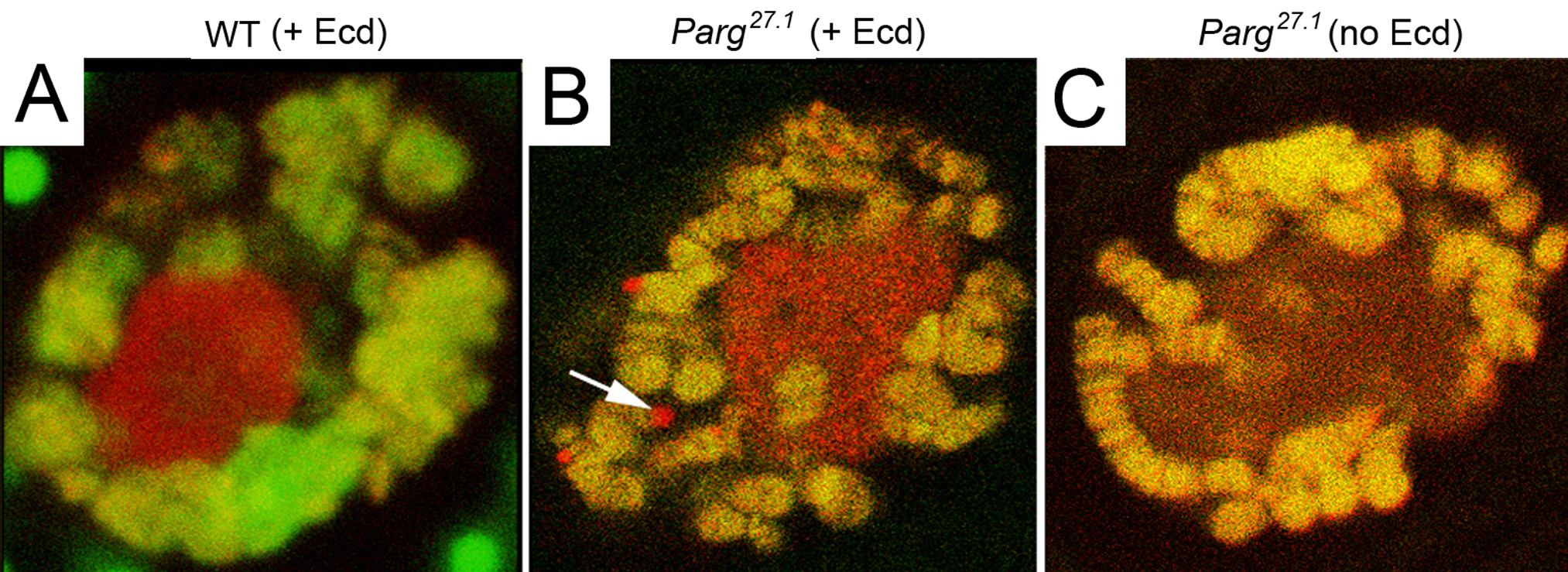

Supplement: Figure S6 — Ecdysteroid hormone controls PARP1 protein localization in vitro. Salivary glands from wild-type (A) and Parg27.1 mutant (B, C), third-instar larvae, expressing PARP1-DsRed (red), were cultured in the presence (A, B) or absence (C) of ecdysteroid hormone (20E), followed by confocal microscopy. DNA was visualized with Draq5 staining (green). Nucleoplasmic PARP-containing bodies induced in Parg27.1 mutant nuclei (B) are indicated with an arrow. N - nucleolus. (3.26 MB TIF) [file pgen.1000387.s006.tif]

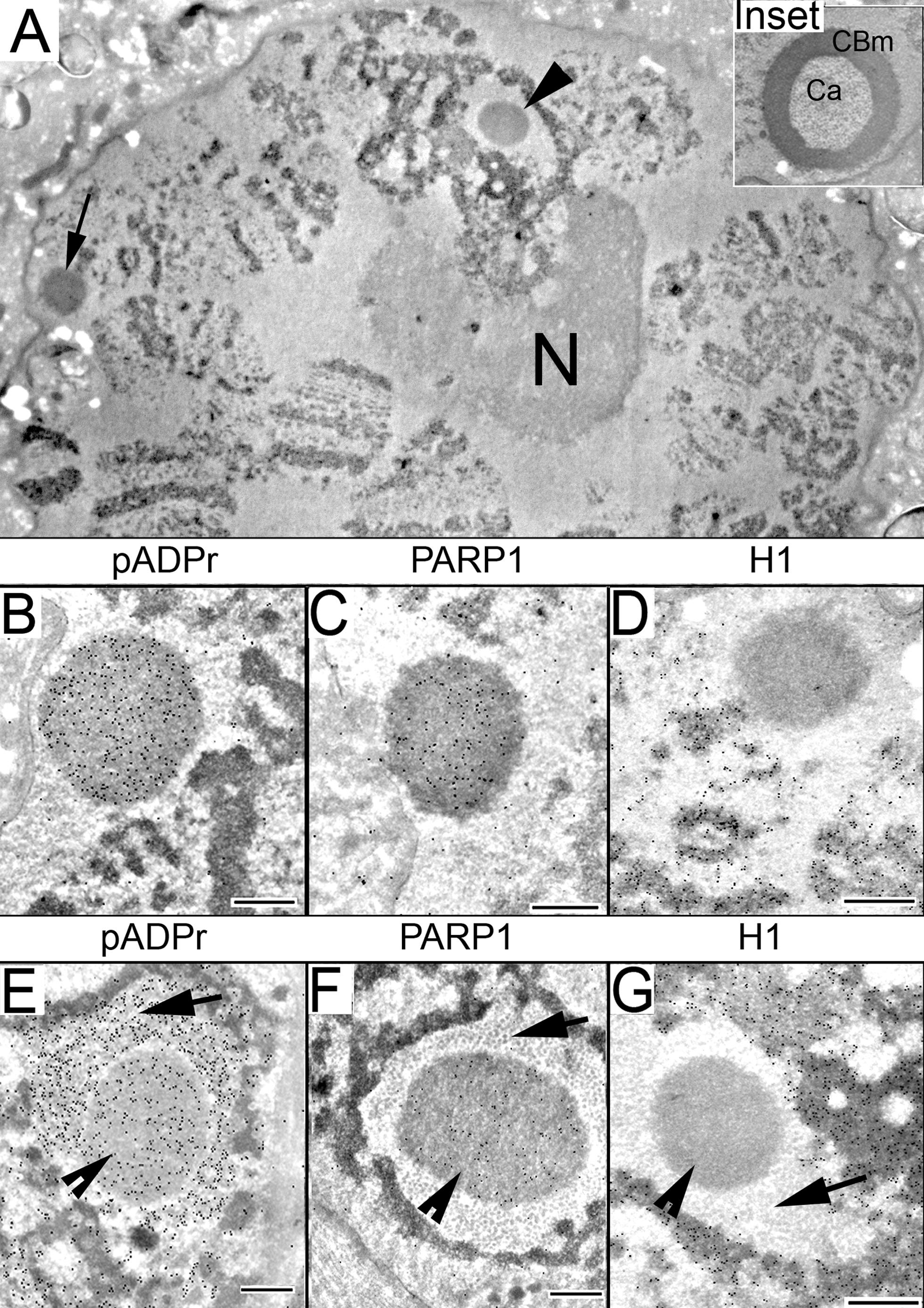

Supplement: Figure S7 — The inhibition of PARG function induces ectopic CBs and relocation of PARP1 protein from chromatin into CBs. (A) The electron micrograph of Parg27.1 mutant nucleus is shown. The “free” nucleoplasmic (arrow) and “chromatin-embedded” (arrowhead) particles are shown. Inset. The cross section through the single CB-like particle is magnified (CBm - Cajal body matrix; Ca - cavity). (B–D) Immuno-gold staining of “free” nucleoplasmic particles using antibodies (1) against pADPr (B), (2) against DsRed (C) and (3) against H1 (D) is shown. (E–G) Immuno-gold staining of “chromatin-embedded” particles using antibodies (1) against pADPr (E), (2) against DsRed (F) and (3) against H1 (G) is shown. Arrowhead indicates CB itself; arrow indicates surrounding materials accumulating on pADPr (E), but not PARP1 (F). (3.98 MB TIF) [file pgen.1000387.s007.tif]

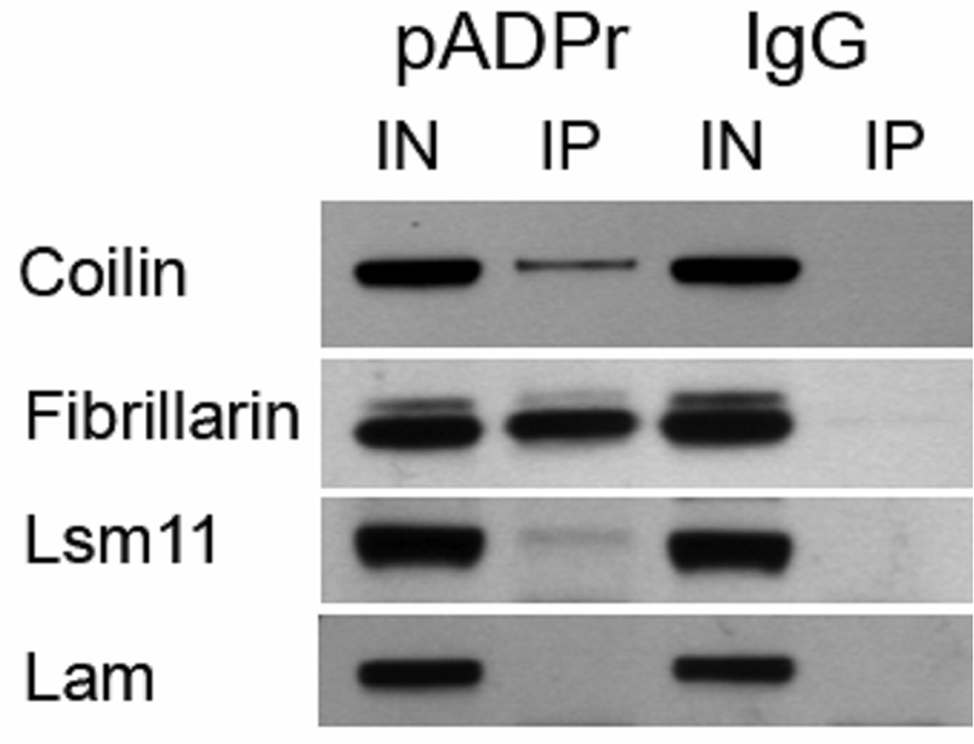

Supplement: Figure S8 — Key proteins of Cajal body and Histone locus body interact with pADPr in Drosophila S2 cells. Co-immunoprecipitation assays using mouse antibody against pADPr (10H). S2 cell culture was used to prepare protein extracts. To detect protein on Western blots, the following antibodies were used: Guinea Pig anti-Coilin, rabbit anti-Fibrillarin, rabbit anti-LSM11 and mouse anti-Lamin C. (0.17 MB TIF) [file pgen.1000387.s008.tif]
